# Engaging men in gender transformative work in institutions of higher learning: A case of the men's hub at Makerere University

**DOI:** 10.3389/fsoc.2022.901049

**Published:** 2023-02-15

**Authors:** Julius Kikooma, Grace Bantebya Kyomuhendo, Florence Kyoheirwe Muhanguzi, Stanley Babalanda

**Affiliations:** ^1^School of Psychology, Makerere University, Kampala, Uganda; ^2^School of Women and Gender Studies, Makerere University, Kampala, Uganda; ^3^Faculty of Social Sciences, Kyambogo University, Kampala, Uganda

**Keywords:** sexual harassment, engaging men, masculinities, gender, social norms

## Abstract

It was noted that globally, sexual harassment (SH), abuse, and exploitation in higher education institutions (HEIs) remain a problem. In Uganda, it regularly made headlines in the media. Yet, it was only after high-profile cases were reported in the media that a spotlight was put on the problem. Moreover, despite there being policies on sexual harassment, changes in reporting processes, and a roster for the swift investigation of sexual harassment cases, sexual harassment persisted in the respective units of Makerere University. The study reported here was based on a project code-named “Whole University Approach: Kicking Sexual Harassment out of Higher Education Institutions in Uganda” (hereafter referred to as the KISH Project). It was action research intended to move beyond feminizing SH interventions and draw in all the key stakeholders with respectively tailored interventions that were need-based. The project applied multiple interventions targeting different stakeholders (including students, academic and support staff, and administrators) to address gaps, prevention, and support for the survivors of SH in HEIs. One of the project components is a “men's hub,” which is aimed at providing space for both male staff and male students to hold dialogs on positive masculinity and call them to act as agents of change in a bid to address sexual harassment within higher education institutions (HEIs). As a platform that brings men together to discuss the issues of sexual harassment, the sessions at the men's hub enhanced their confidence and ability to prevent and respond to sexual harassment as well as their knowledge about the issues of masculinity and how they relate to sexual harassment. It was found to be an empowering platform with opportunities for awareness creation and the potential for amplifying the role of men in influencing change by speaking up and acting on their masculinity to address sexual harassment.

## 1. Introduction

Sexual harassment (SH), abuse, and exploitation remain a problem that has been widely reported in workplaces (Mukoboza, 2016). In fact, there is a wide range of literature on this subject (Pyke, 1996; Berman et al., 2000; Thomas, 2004; Huerta et al., 2006). It indicates that sexual harassment is a key issue at higher institutions of learning and is a real problem, which affects students' health, emotional wellbeing, and ability to succeed academically (Hill and Silva, 2005). Some of the literature makes the point that although students are disproportionately affected (due to the hierarchies in the power dynamics of the student-teacher relationship), everyone in this university campus setting is a potential victim of sexual harassment since female students, female lecturers, and male students are all harassed. The question of hierarchies in the power dynamics at these institutions especially as it relates to the social construction of gender is treated as a key cause for the victimization, not only of female students but also of female faculty, especially when compared to the victimization that happens against men. According to the Ugandan National Academy of Sciences study of 2018, women are much more likely to be the victims because they are often more vulnerable than men. The reasons cited are their lack of power which puts them in more vulnerable positions. Nonetheless, men too can fall victim to both male- and female-perpetuated violence in university settings (Namitala, 2022).

Engaging men has now become part of established global efforts to prevent violence against women and girls (VAWG; Flood, 2011; Chakraborty et al., 2016, 2018), which is widely recognized as a pressing social issue across the world and is considered a violation of basic human rights, as well as having adverse effects on social, political, and economic inequity. Indeed, there are growing efforts globally to involve boys and men in the prevention of violence against girls and women. Efforts to prevent violence against girls and women now increasingly take it as a given that they must engage men and, as Flood (2011) noted, men are called upon to be involved in a range of initiatives as participants in education programs, as targets of social marketing campaigns, as policymakers and gatekeepers, and as activists and advocates. Most interventions focus on making men's behaviors and attitudes more gender equitable.

Issues of gendered violence and inequity have been addressed most prominently by feminist scholarship and research since the 1970's, which have recognized and highlighted the multilayered nature of such violence expressed in the multifaceted and hierarchical relationships between and among groups of men as well as between men and women. The direction taken in the literature that supports such scholarship argues for charting a path for allyship that capitalizes on the possibilities of moving from violence to supportive practice (Chopra, 2003; Flood, 2015). Scholars who hold this perspective argue that social research should take seriously men's current supportive practices (Cash and Smith, 2010; Macabre, 2012; Casey et al., 2016). While scholarship on male allies has demonstrated the nature of their transformations and motivations, less attention has been paid to their negotiations of masculinity, privilege, and the intersection between subjecthood and social contexts.

Feeding into conversations on how sociocultural and political constructions of hegemonic masculinity and patriarchal social norms underpin violence against women and other gendered bodies at interpersonal and structural levels, the relatively new and rapidly expanding research field of Masculinity Studies or Critical Men's Studies calls for deeper understandings of the social contexts in which men become engaged and the shifts, negotiations and changes that such work engenders in their conceptions of masculine identity subjecthood and community (Christofidou, 2021). As Christofidou (2021) noted, the debates currently unfolding in the field of critical men and masculinities studies concerns whether and how men and masculinities are changing and how these inform their engagements with women activists' anti-violence work in their communities. Such studies insist that men and masculinities be recognized as critical, both socially and politically, and foreground some of the ways in which particular versions of masculinity were and remain a key problem globally (Connell, 1998, 2000, 2001; Kimmel et al., 2005; Hearn et al., 2006). There is also a growing focus on men and masculinities in research on the African continent as well (Ratele, 2006, 2016; Shefer et al., 2007; Clowes, 2013; Mwine, 2018, 2019, 2020; Ahikire and Mwine, 2020). Alongside studies on gender, violence, and sexuality studies focusing on masculinities in Africa, scholars have begun to explore the social construction of heterosexual masculinities, particularly between and among young men and boys on the continent.

However, much of this research has been inspired by the imperatives of challenging the spread and impact of HIV/AIDS in Africa, and such research has inadvertently tended to demonize boys and men, constructing them as inherently problematic. As a result, such scholarship has not always provided a more nuanced picture of the complexities of the social construction of masculinity and the often contradictory experiences of boys and men (Pattman, 2007; Shefer et al., 2007; Bhana and Pattman, 2009). Relatedly, there is relatively little documentation on how programs that engage men and boys in the prevention of violence against women may lead to change or reformulation of masculinities in the African context. In feminist-informed programs, critically examining traditional assumptions about gender, and particularly masculinity, constitutes a central component of discussions with men regarding dismantling violence (Casey et al., 2012; Dozois and Wells, 2020). A number of interventions in this line of thinking invite men to reimagine closely held beliefs about their own gender but with the infrequent theorization of how the desired change can be supported to occur. As Gibbs et al. (2015) pointed out, the lack of an explicit theory within interventions working with men and boys means that it is sometimes unclear what change is sought.

### 1.1. Fighting sexual harassment in HEIs in Uganda

Addressing gender-based violence in all its manifestations is a priority area highlighted in a number of the Government of Uganda policies and plans including the 2015 National Development Plan (NDPII), the 2016 Social Sector Development Plan, the 2016 Gender Based Violence policy, and the 2007 Gender Policy. Notwithstanding the national level of commitment *via* the supportive policy and legal framework, the government recognizes that it has not fully addressed the problem. In a report of the parliamentary select committee on an inquiry into the allegations of sexual harassment in institutions of higher learning in 2019, the committee found that sexual violence is widespread in virtually all the institutions of learning visited. The committee however noted that the actual prevalence of the vice in the country is difficult to determine as many of the cases are never reported. It was also observed that in Uganda, there is no sufficient empirical information to initiate targeted programs for empowering students and staff to respond to and prevent sexual harassment in HEIs. Among other recommendations, the committee charged the Ministry of Education and Sports with formalizing the collection of data on sexual violence in the institution of higher learning and publishing it on an annual basis.

While academic institutions are considered elitist spaces with high awareness levels and intellectualism (Nyende, 2006 cited in Namitala, 2022), sexual harassment has received unprecedented attention in recent years within academia. There have been opportunities to question hegemonic beliefs and practices upon which harmful forms of masculinities in higher education institutions are constituted through masculinities scholarship. Opportunities have also increasingly opened up to research men's experiences including how masculinities and femininities relate to the reproduction of inequalities. Moreover, while the drivers of sexual harassment are related to a range of underlying factors, it is part and parcel of manifestations of gender-based violence and the abuse of power. To address the challenge, Makerere University has put in place a policy and regulations against sexual harassment to curb the vice within its structures. The *Policy and Regulations Against Sexual Harassment* at Makerere University defines the term “sexual harassment” as “unwelcome sexual advances, requests for sexual favors or unwanted physical, verbal or non-verbal conduct of a sexual nature.” To date, the university has a number of policies, systems, and structures for addressing sexual harassment at all levels including the Gender Equality Policy and the anti-sexual harassment policy objectives of ending sexual harassment, as well as the Gender Mainstreaming Directorate as a cross-cutting measure in all its operations. Yet, despite those structures, sexual harassment persists in the respective units of Makerere University (Namitala, 2022). It has also been noted that several cases go unreported, and there is a culture of fear and silence among the victims of the abuse (Makerere University, 2018; Namitala, 2022).

### 1.2. The problem

As part of the response to the above situation, Makerere University revised its policy on sexual harassment, changed reporting processes, and created a roster for swiftly investigating sexual harassment cases. The measures aim at imposing strict punishments for any infraction of the rules. However, there is a danger with this approach as these conflate behaviors and do not help in understanding or tackling the roots of unacceptable actions. Dysfunctional systems are not changed through “quick fix” punishments of offenders nor through codes of conduct and disciplinary procedures. As Alison Phipps (2017) pointed out, this is called “institutional airbrushing.” What you get is that the visible blemish is removed and the underlying malaise is left to fester. Tackling sexual harassment on campus is more than naming and shaming. As others (Huerta et al., 2006; Mukoboza, 2016) have argued, seeking justice for sexual harassment without acknowledging the injustices built into the fabric of institutions will protect some at the expense of others. Without wider cultural change, they can simply create compliance through fear of punishment, which is the opposite of systemic reform. What is needed is cultural change, which makes a difference in the long term.

Against that background, the School of Women and Gender Studies working in collaboration with the School of Psychology, the School of Computing and Information Technology, and Kyambogo University undertook to provide a solution to the persistent sexual harassment within higher education institutions. The proposed solution was the project titled “*Whole University Approach: Kicking Sexual Harassment out of Higher Education Institutions in Uganda*” *(KISH Project)*. The KISH project was implemented with support from the Government of the Republic of Uganda through the Makerere University Research and Innovations Fund (Mak-RIF). The overarching objective of the project was to provide holistic measures involving all key stakeholders and institute multiple interventions targeting different stakeholders with different needs and capabilities.

The adoption of a holistic approach was one of its unique selling points for a number of reasons. First, it had been noted that the existing interventions were largely reactive in nature, mainly focusing on investigations of reported cases. This was already a serious constraint, given the parliamentary select committee findings that indicated that several cases go unreported and the existence of a culture of fear and silence among the victims. Second, the existing structures that include SH committees at various administrative units were largely inactive with no clear direction of work. Third, the focus on students was predominantly through sensitization meetings. Fourth, there had been no program specifically targeting male students and staff on the issues of sexual harassment in higher education institutions in Uganda.

### 1.3. The men's hub at Makerere University

One of the KISH project components is a men's hub for male staff and male students to dialog on masculinity, their roles, and accountability on issues of sexual harassment. It is a space created for a men's “hangout” club to hold fireplace-type conversations among men's groups around gender and violence against women and girls linking them with women's situations in their environment. It is designed to complement traditional “changing minds” approaches to behavior change (e.g., psychoeducational programming) using environmental cues to increase prosocial, equitable behaviors in specific male-oriented settings.

Through the KISH project, the men's hub started engaging men in the drive to end sexual harassment by forming men's groups on campus on a pilot basis. The pilot has been used to develop some materials for training peer groups in the prevention and engendering accountability among males engaged in programs for ending sexual harassment, abuse, and exploitation in higher education institutions in the country. At the moment, work accomplished has been undertaken at Makerere University. In the second phase of the project, it was replicated at another public university: Kyambogo University. The plan was to scale it to other institutions of higher education.

The intention was to come up with a platform that would enable us to create the social conditions that will stop SH before it starts. The idea was to work with men to cultivate capacities in three key areas for gender transformation, namely, gender equality, healthy masculinities, and healthy relationships. The hub's activities are geared toward strengthening this combination of capacities in “constructed” male-oriented settings with the hope that this would lead to healthy relationship competencies to stop the perpetration of multiple forms of gender-based violence including intimate partner violence, violence against women, dating violence, and peer-to-peer aggression. Approaching SH through a focus on male-oriented settings helps in amplifying the signals associated with gender equality and healthy relationships and correspondingly works to disrupt signals related to inequality, discrimination, and violence. Ultimately, the men's hub is working to support men to move toward becoming agents of change in public and observable ways.

Core themes from the conversations include paths to masculinity, the transmission of masculinity, male understandings of women, and the emotional lives of men. Paths to and transmission of masculinity deal with the participants' experiences and reflect on the ways in which they experience and learn about masculinity and where they derive these experiences and lessons. Male understandings of women capture the participants' views of women and how they relate to them in their everyday life. The emotional lives of men and transitional moments describe the participants' emotional experiences and the ways that these experiences facilitate meaningful changes in their lives.

## 2. Research framework

The KISH approach is a holistic system to prevent and respond to sexual harassment (SH) through student-friendly innovations that address knowledge gaps, prevention, and support for the survivors of SH in HEIs. It was action research intended to move beyond feminizing SH interventions and draw in all the key stakeholders with respectively tailored interventions that were need-based. The project included four key components, namely, an online KISH system for reporting, supporting, and processing cases of sexual harassment (SH) in a confidential and safe space; KISH Student's Clubs for capacity building in life skills and knowledge about prevention and reporting systems for SH for undergraduate female students which are managed by the trained coaches; online SH course for providing information on SH, policies and structures for addressing SH, sources of support, and response mechanisms for staff and students; and the men's hub for providing space for male staff and students to hold dialogs on positive masculinity and encouraging them to be the agents of change as a strategy for addressing SH within in HEIs.

### 2.1. Materials and methods

As an action research, the study utilized a mixed-methods approach involving evaluation methodologies to establish the impact of the proposed innovations. This included undertaking a baseline survey for establishing the characteristics, perceptions, and experiences of SH, knowledge of prevention and response mechanisms, user needs, and expectations of the KISH online application and course. An end line survey to establish the impact of the interventions was planned for but the results reported here are based on a midterm evaluation that was carried out after one year of implementation. Data were collected using the quantitative and qualitative methods for both the baseline and end-line surveys, respectively. Quantitative data were obtained using a questionnaire for students. Qualitative data were generated through key informants and in-depth interviews and focus group discussions for students and staff. Given the multiple components constituting the whole project, specific activities and results relating to each component were reported separately. Since the focus of this study was on the issue of engaging men in the fight against sexual harassment, the rest of the sections will be on the experience from this project relating to the men's hub component.

The mode of operation of the hub involves holding men's dialog groups for students and staff established as the nodes of conversation; quarterly meetings/seminars of certified hub members/alumni on different male-related topics; joint meetings for staff and students held once a year to showcase solidarity and their role in promoting masculinity; and an annual event of men's dialog to share with the public. We conducted 15 men's dialogs with groups of students and staff totaling 288 (i.e., 173 male students and 115 male staff). The workshops introduced participants to the following: sexual harassment, its forms, and effects; the concept of masculinity; the link between masculinity and sexual harassment; and progressive forms of male behavior that promote a sexual harassment-free learning environment.

As explained earlier, the men's hub involves holding dialog sessions in a workshop format targeting male staff and students, focusing on harnessing positive masculinity to address SH and sharing knowledge on SH, and developing strategies as a group to stop SH in HEIs. The workshops mainly focus on the terms “male sexuality” and “masculinity” as the descriptors for the socialization process that influence male sexual expression. This is after realizing that the manner in which the socialization processes are embedded within the men's sexuality and their psyche is ill-explored. Frequently, we got feedback from the participants pointing to the fact that the socialization processes that men undergo in their sexual development can lead them toward normalizing sexual violence.

Analysis of masculinity at the men's hub focused the attention of participants on the ill effects hegemonic masculinity has on men, as well as the detrimental and disastrous effects that it has on women. We engaged experts to help us reflect on the genealogy of its ill effects. The participants were asked to reflect on how they socialized into being men in their contexts. They reflected upon who their agents of socialization were that influenced their masculinities (i.e., the categories of masculinity they developed). These reflections were conducted using reflective tools, such as the “manbox,” to recognize the challenges men face in trying to fulfill society's expectations about gender roles and the cost of harmful masculinities and to develop new perceptions for change.

After 1 year of implementation, a mid-term evaluation was conducted. The main objectives of the evaluation were to (1) track progress in the implementation of the project after 1 year; (2) identify the achievements as per the project objectives and outcomes; (3) examine the stakeholders' perceptions regarding the intervention's ability to address sexual harassment; and (4) identify the strategies for the uptake and sustainability of the interventions. The evaluation targeted male students and staff who participated in the men's hub activities and their facilitators. It focused on knowledge acquired about SH, prevention and response, positive masculinities and life skills, and the application of the knowledge and skills to address SH.

## 3. Results

The dialog sessions used the opportunities for reflection provided by the hub's environment to get participants to reflect on the importance, not only of acknowledging the negative impact that dominant forms of masculinity have on women but also on how they undermine boys' and men's health and wellbeing. In addition, this awareness—of the dangers of conforming to harmful masculine lifestyles—was the basis for engaging the participants to question and seek to transform harmful practices. The key findings from the sessions are organized under three issues, namely, men's understanding of sexual harassment; the causes of sexual harassment; and what men stand to gain from gender equity and a violence-free academic environment.

### 3.1. Understanding sexual harassment

Regarding the manifestations/forms of sexual harassment, men's perception reflected a narrow understanding of sexual harassment at the beginning of the dialogs. Most of the men were limiting sexual harassment to sexual intercourse, leaving out many other forms. However, as the dialogs continued and heated debates arose, more forms and manifestations were identified and these helped in widening and deepening their understanding of sexual harassment as exemplified in the following extracts describing it as:

*Inappropriate physical conduct of any body parts such as scratching, pinching, stroking or brushing up the body*

*Unwanted and persistent explicit or implicit propositions to engage in sexual activity*

*Intentional disrobing or exposure of sexual body parts or underwear*

*Unwanted demands for sexual relations in exchange for employment or academic or other favors*

*Sexual scares such as leering and ogling with suggestive overtones*

*Lustful gestures such as hands or sign language to denote to sexual activity*

*Stalking through following or spying on a person*

*Sexual assault and rape*

*ICT or Cyber based sexual harassment such as trolling through tweets, text messages and sharing unwanted private massages and photos on social media platforms*.

The male staff participants argued that the existing policy against sexual harassment at Makerere University seems to be narrow in defining what comprises sexual harassment and that it needs to be widened and made more clear on what constitutes sexual harassment. Having a broader understanding of sexual harassment would help them to avoid operating in ignorance.

### 3.2. Causes of sexual harassment

With regard to the causes of sexual harassment, different participants come up with different accounts. They revolved around the nature of socialization the men go through and the culture of silence.

#### 3.2.1. The nature of socialization the men go through

Among the commonly agreed upon causes was the nature of socialization that men go through in the various social institutions that make the boy child develop a feeling of sexual entitlement at the expense of the girl child. Besides, power games in the form of power over women that men mainly use to exploit women sexually were also mentioned. One of the examples that come out vividly was the power of the red pen held by the lecturer.

“*With the power of the red pen in the hands of the male lecturer who feels entitled to sexual pleasure, the young girls around campus can hardly survive sexual harassment*” (a male student participant).

Most of the participants directly or indirectly linked sexual harassment to the long-term effects of one's possession of toxic masculinities. The participants argued that they or their male friends exhibit behaviors that might result in sexual harassment. Such acts include the following: use of threatening tone or words, aggressiveness, desire to control others using hands, suppression of emotions, treating sex as a competition, men talking about their conquest, feeling entitled to sex every time you see a woman, looking at women as sexual objects, looking at women as weak, and categorizing some men as weaker because of their sexual behavior mainly those with one sexual partner.

Related to toxic masculinities, male students discussed the issue of the ideal male student behavior at university and how it related to sexual harassment. Male students argued that the ideal male student and sexual harassment are related. They insisted that they are about

“*Be having many girls or ‘importing' or sleeping with many of them,”*

“*getting ladies for sex at all costs,”*

“*being physical fit it which includes the ability to sleep with a number of girls at campus,”*

“*being vulgar, we observe men at university taking pride in throwing vulgar words to ladies,”*

“*being intelligent in class and discussions makes more girls attracted,”*

“*a drunk male student would have a strong desire to sexually abuse their female counterparts*.”

Male student participants conceded that the normalization of such behaviors referred to as ideal male student conduct at university has rendered many male students to become seasonal perpetrators of sexual harassment.

They, however, suggested that the only way to overcome this challenge is through promoting progressive masculinities because, for them, there is no relationship between being a gentleman and sexual harassment. Being a gentleman demands that you will have one girlfriend, will respect the ladies, and will try as much as you can to support the ladies to settle on campus and progress academically.

Upon realizing that conforming to what is commonly understood in campus language as “cool masculinities” has the potential to institutionalize sexualized relations, the hub participants noted that this is the recipe for the toxic masculinity practices that lead to SH. These “cool” albeit toxic masculinities are characterized by,

*Looking nice—gals fall for you “Importing” Having sex with multiple girls, Drinking/clubbing Braving the strike, Sex, just-for-just Updating sexual relations, and Vulgarity (Mwine, A. Men's Hub facilitator)*.

Conforming with such sexualized masculinities comes with a cost, not only in financial and health terms, but also in terms of lost time for education (inadequate attention and poor academic performance), negative institutional reputation (unsafe and sexualized spaces), and essential learning in institutions of higher education about sexual relationships and legitimizing a subtle culture of sexual oppression. In addition, participants also decried the general decay of moral values accompanied by diverse cultural differences among people. This came out during a heated debate on whether a dress code that is perceived as indecent can cause sexual harassment or not. On this issue, dialog participants would identify with either of the two positions. One position was a view that perceived indecent dressing was responsible for tempting men to sexually harass their female counterparts, given the way the women dressed. However, the second position was based on a disagreement with the issue of perceived indecent dressing as a justification for sexual harassment. They argued that different cultures define indecency differently and that what one considers indecent among the Baganda for instance might not be indecent among the Karamojong. The Baganda people constitute the largest ethnic group in Uganda. The Karamojong people are an ethnic group that lives in the northeastern part of Uganda and have a very different cultural setup from that of the Baganda people.

#### 3.2.2. Culture of silence

The participant's views on reporting sexual harassment cases were negative, indicating that the majority of men never believed in reporting sexual harassment cases whether as victims or bystanders. During the dialogs, male staff and students defended their unwillingness to report sexual harassment raising arguments like:

- “*We were advised that we use counseling to help the ‘student harassers' rather than run out of class...”*
- “*Oh, imagine a male lecturer reporting a female student for sexual harassment! Of course I have been a victim but.... it's just tricky.”*
- “*I was harassed and asked a female lecturer colleague to help and counsel the student. It was tricky too.”*
- “*By the way forms and motives for harassment differ and the response/management needs to be response specific*.”
- “*Men are quiet because they are just reserved, and take it normal*.”
- “*It is fairly easier to survive an ‘attack' by a student on the part of a male lecturer who is not ready to fall victim than it is when a student is harassed by a lecturer*.”
- “*The issue of SH has been largely one sided, always portraying men as perpetrators while women are always victims… so, I think the chances of my claims being taken seriously/believed are low. Perhaps I will be seen as not being ‘man enough*.”'
- “*Some students feel very beautiful and think they can lure any man. They therefore feel the power to move male lecturers by their sexual attractions*.”
- “*Although there have been cases where a female harasses a male but when the male refuses, the female say, am going to shout that you want to rape me and so, from harassment to blackmail and threats.”*
- “*However, on the other hand, men are not empowered to report violence against them. The superiority complex shuts them down and suffer with the abuse.”*

Both male students and staff alluded to the challenge of men reporting sexual violence as having structural and psychological constraints in that nobody in the concerned offices seemed to believe men can be sexually harassed. To them, society has been made to believe that it is men who harass and not otherwise. Below, we share some notable quotes from the men's conversations at the hub:

...The issue of SH has been largely one-sided, always portraying men as perpetrators while women are always victims. So, I think the chances of my claims being taken seriously/believed are low. Perhaps I will be seen as not being “man enough”...

...Some students feel very beautiful and think they can lure any man. They therefore feel the power to move male lecturers by their sexual attractions...

...A female student comes “Help me I am willing and ready to do anything”...

Credibility and victim-blaming were other causes that participants pointed out. According to the participants, the more victims are blamed for having been sexually harassed, the more they shun away from reporting and seeking help and this gives leeway for the perpetrators to continue. They also hinted that most of the existing structures make it a blame game by asking the victims questions such as:

“*What were you doing in his room? Why did you walk alone at night? Why did you dress like that?*”

Such blame game-based questions and others tend to scare away victims from reporting cases of sexual harassment, and this causes the persistence of the vice.

Regarding the participant's perception of who are the most common victims of sexual harassment, they indicated that

“*It could be anybody, all of us, anybody can be a victim, Staff and students, mainly female students, male and female, both gender, but I think the first party to perceive it, mostly those with least power in a relationship, students can be perpetrators of sexual harassment against male lecturers, even male students against female lecturers*.”

### 3.3. What men stand to gain from gender equity and a violence-free academic environment

The responses of men's hub participants during the midterm evaluation indicated that they appreciated the opportunity that the men's hub provided. They noted that it helped them to engage with real-life situations as men, re-learn, and unlearn from each other as well as getting to understand how they contribute to sexual harassment. In addition, they observed that the dialogs provide them with information to make informed decisions about their behaviors and “control themselves from falling into trouble,” other than traditional culturally held notions of masculinities and of who a man should be.

Below are some men's hub voices from the respondents who participated in the midterm evaluation.

*The strength of the hub is getting men to talk to each other and with each other because this is a subject that is rarely got before men to discuss and talk about, so I think that's the strength of the hub. When men normally meet, they tend to talk about other things but not issues of sexual harassment of the women. (IDI, Men's Hub—Staff)*

*The men's hub has given information and taught us about sexual harassment online, how to report and how to prevent ourselves from being perpetrators of the sexual harassment vices more so it has enabled us to reach out to the students with such information because we have held several meetings hence its meeting these objectives. (IDI, Men's Hub—Student)*

*Where I work, I deal with gender-based violence, I have done research on gender-based violence, I have had an addition knowledge, the concept of positive masculinity I knew of, and I thought was in practice, but I did not know what I was practicing. I knew it was all about being fair, I did not know about the concept of positive masculinity. That has to be a lesson and a course that has to be taught to others. (IDI, Men's Hub—Student)*

According to these students and staff, the sessions enhanced their confidence and ability to prevent and respond to sexual harassment as well as their knowledge about the issues of masculinity and how they relate to sexual harassment. The men's hub as a platform that brings men together to discuss the issues of sexual harassment was found to be an empowering platform with opportunities for awareness creation, amplifying the role of men to influence change by speaking up, and acting on their masculinity to address sexual harassment.

In total, five specific benefits for men's hub participants were identified for promoting an educational environment that is free of SH. These include the following:

1. Academic excellence—young men performing exceptionally well. This can be made through intentional processes of challenging and re-imagining masculinities on the campus that lead to crafting new values, rules, and expectations of being a university student—(e.g., celebrating academic performance), and they can act as partners in the fight against SH in the university by:
  a. Promoting positive masculinities which are progressive in nature.
  b. Supporting their female counterparts in progressively pursuing their academic and career goals.
  c. Treating women the way they would have treated their sisters or daughters.
  d. Sensitizing their male counterparts on the need for creating a favorable environment for working and learning that is free of sexual harassment threats.
  e. Understanding that women are not sub-humans, they can suffer other people's conduct against them, and they need to realize that these are students and not sexual objects. They need to be treated as students who need to be helped to improve their performance. … *We need to treat these students as our daughters*.
  f. Reconceptualization of women not as sexual tools.

2. Hub members identifying, reporting, and condemning cases of SH.
3. Mentor each other and new university entrants on non-violence and SH-free practices.
4. Open up opportunities to question hegemonic beliefs and practices upon which harmful forms of masculinities in higher education institutions are constituted to include deliberately engineering unlearning, and re-learning of everyday cultural expectations that compel men into SH.
5. Participating in university leadership; including mentorship of freshers—student leaders.

## 4. Discussion

When the men's hub was set up at Makerere University, it sought to operationalize a conceptual model for engaging men as advocates for change, as advanced by Funk (2018), centered on working with men to build capacities in the following three key areas: gender equality, healthy masculinities, and healthy relationships. During the dialogs, male students and staff members proposed a number of ways through which they can act as partners in the fight against sexual harassment at the university. These included the following:

- Having a broader understanding of sexual harassment so as to avoid operating out of ignorance.
- Embracing reporting of cases of sexual harassment whether as victims or as bystanders.
- Practicing morality and ethical values as men in society.
- Talking to students openly to reveal to them that none of the two parties is meant to harass the other sexually.
- Breaking the silence about sexual harassment.

Taking responsibility for their personal actions and views regarding matters of sexual harassment such that… “we stop blaming one another but ourselves.”

As the work evolved, the emphasis shifted to include a greater focus on social norms and networks. It was realized that engaging a socially privileged group in dismantling structures that benefit them can be challenging. Indeed, during the activities of the men's hub, two issues become manifest during critical reflections between participants and the facilitators. First, as Casey et al. (2012) pointed out critically exploring traditional masculinity and its associated privileges generates one of the fundamental tensions inherent in engaging men in anti-violence work. It is like inviting them to change closely held beliefs about their own gender. It is tantamount to asking them to shed the privileges that accrue to them based on gender. Therefore, while participants appreciated the benefits of a more gender-equal society, they also realized they had something to lose from restructuring the prevailing systems of power. So, while all men's hub participants were initially interested to be part of the call to men to be agents of change, many were cautious or hesitant as some of the extracts presented under the results on men's socialization and the culture of silence indicated. This goes to show that when mapped on Funk's (2018) three rungs continuum of male engagement to prevent gender-based violence and promote gender equality, the majority were at the “maintain status” quo or the “aware” rungs. “Maintain status” participants were characterized by hostile, opposed, resistant or uninterested views, and attitudes toward promoting gender equality. Participants' views categorized under the “aware” rung were hesitant, curious, and interested. In all, both categories fall below the ultimate rung on Funk's (2018) continuum of male engagement labeled “advocate” where one demonstrates the characteristics of being inspired, engaged, influencing and even leading efforts to prevent gender-based violence and promote gender equality.

Second, traditionally toxic masculinities are associated with gender inequality and violence against women. Yet that framing is based on a deficit model (i.e., tells men what they should not do, rather than focusing on alternative attitudes and behaviors) that reinforces a simplistic gender binary (i.e., man/woman) with no scope for flexibility, reflection, or experimentation and as such passively contributes to the reproduction of patriarchal relations and structural inequality. What emerged from the excerpts of participants reflecting on what men stand to gain from gender equity and a violence-free academic environment indicates that a shift in emphasis is needed to tap the power of networks as cornerstones for transmitting cues for human behavior change. According to Dozois and Wells (2020), human behavior is governed by a subtle but pervasive set of cues that are transmitted through networks. It is therefore plausible to suggest that male-oriented settings have the potential to amplify the signals associated with gender equality and healthy relationships. The efforts of the men's hub in this respect are geared toward disrupting signals related to inequality, discrimination, and violence and building their capacity to help each other so that people in those settings can increasingly engage in healthier and equitable relationships.

## 5. Conclusion

Using experiences from an action research intervention project on engaging men and boys in gender transformative work, the article interrogates what men can do to work with women in challenging the institutionalized nature of sexual and gender-based violence. Extracts from the men's dialogs and their responses from the mid-term project evaluation suggest that men are interested in the work, but they are also hesitant or cautious. Assessing ways men respond, react, or behave in this kind of work and recognizing where they are at in the process can help in supporting men's transition into advocates for change.

## Data availability statement

The raw data supporting the conclusions of this article will be made available by the authors, without undue reservation.

## Ethics statement

The studies involving human participants were reviewed and approved by Makerere University, Social Sciences Research Ethics Committee. The Ethics Committee waived the requirement of written informed consent for participation.

## Author contributions

JK, GK, and FM contributed to conception and design of the study. SB organized the database. JK wrote the first draft of the manuscript. All authors contributed to manuscript revision, read, and approved the submitted version.
